# Lymphoepithelial Carcinoma of the Lung: A Case Report and Review of the Literature

**DOI:** 10.7759/cureus.70309

**Published:** 2024-09-27

**Authors:** Menelaos G Samaras, Nektarios Ι Koufopoulos, Sofoklis Mitsos, Eris Dylja, Athanasia Monokrousou, Periklis Tomos, Ioannis G Panayiotides, Dimitrios Goutas

**Affiliations:** 1 Department of Pathology, School of Medicine, Attikon University Hospital, National and Kapodistrian University of Athens, Athens, GRC; 2 Department of Thoracic Surgery, University College London Hospitals NHS Foundation Trust, London, GBR; 3 Department of Surgery, School of Medicine, Attikon University Hospital, National and Kapodistrian University of Athens, Athens, GRC; 4 Department of Thoracic Surgery, Attikon University Hospital, National and Kapodistrian University of Athens, Athens, GRC; 5 Department of Thoracic Surgery, School of Medicine, Attikon University Hospital, National and Kapodistrian University of Athens, Athens, GRC

**Keywords:** ebv infection, lung cancer, lymphoepithelioma-like carcinoma, non-small cell lung cancer, squamous carcinoma

## Abstract

Lymphoepithelial or lymphoepithelioma-like carcinoma is a poorly differentiated carcinoma located outside the nasopharynx with similar morphologic characteristics to its nasopharyngeal counterpart. Lymphoepithelial carcinoma of the lung is a rare subtype of squamous cell lung carcinoma frequently associated with Epstein-Barr virus (EBV) infection, accounting for approximately 1% of non-small cell lung carcinomas (NSCLC). We herewith present a case of a 78-year-old female patient who was diagnosed with lymphoepithelial carcinoma of the lung, emphasizing its distinct epidemiological features, clinical workup, and histopathological characteristics. Furthermore, we discuss its histologic differential diagnosis. Finally, we refer to this tumor's unique molecular and immunological profile and its treatment modalities, and we review the literature.

## Introduction

Lymphoepithelial carcinoma of the lung is a rare entity, first described by Begin et al. in 1987 [1]. It is classified in the 2021 WHO classification update as a poorly differentiated squamous cell carcinoma, infiltrated by variable amounts of lymphocytes and plasma cells, frequently associated with Epstein-Barr virus (EBV) [2]. It accounts for approximately 1% of non-small cell lung carcinomas (NSCLC) and mostly affects people of Asian origin, non-smokers, with a median age of 51 years [3]. Histologically, it is similar to its sinonasal counterpart and typically shows a syncytial growth pattern with cells showing vesicular nuclei and distinct nucleoli, variably abundant eosinophilic cytoplasm, a variable number of mitotic figures, and diffuse interstitial lymphoplasmacytic infiltration [3]. Its diagnosis may be challenging, especially on frozen sections, due to its morphological similarity to non-keratinizing squamous cell carcinoma, pulmonary nuclear protein in testis (NUT) carcinoma, and non-Hodgkin lymphomas [4]. Similar carcinomas have been reported in various anatomic sites, including the breast [5, 6], gastrointestinal tract [7, 8], hepatobiliary system [9], kidneys [10], major and minor salivary glands [11, 12], thymus [13], thyroid [14], urinary bladder [15], and uterine cervix [16]. Depending on the stage at diagnosis, therapy consists of complete resection combined with chemotherapy/radiotherapy (neoadjuvant or adjuvant) or immunotherapy [4, 17]. Survival rates are higher compared to conventional NSCLC [17]. We herewith present a case of lymphoepithelial lung carcinoma with distinct clinical and epidemiological features and a literature review.

## Case presentation

**Figure 1:**
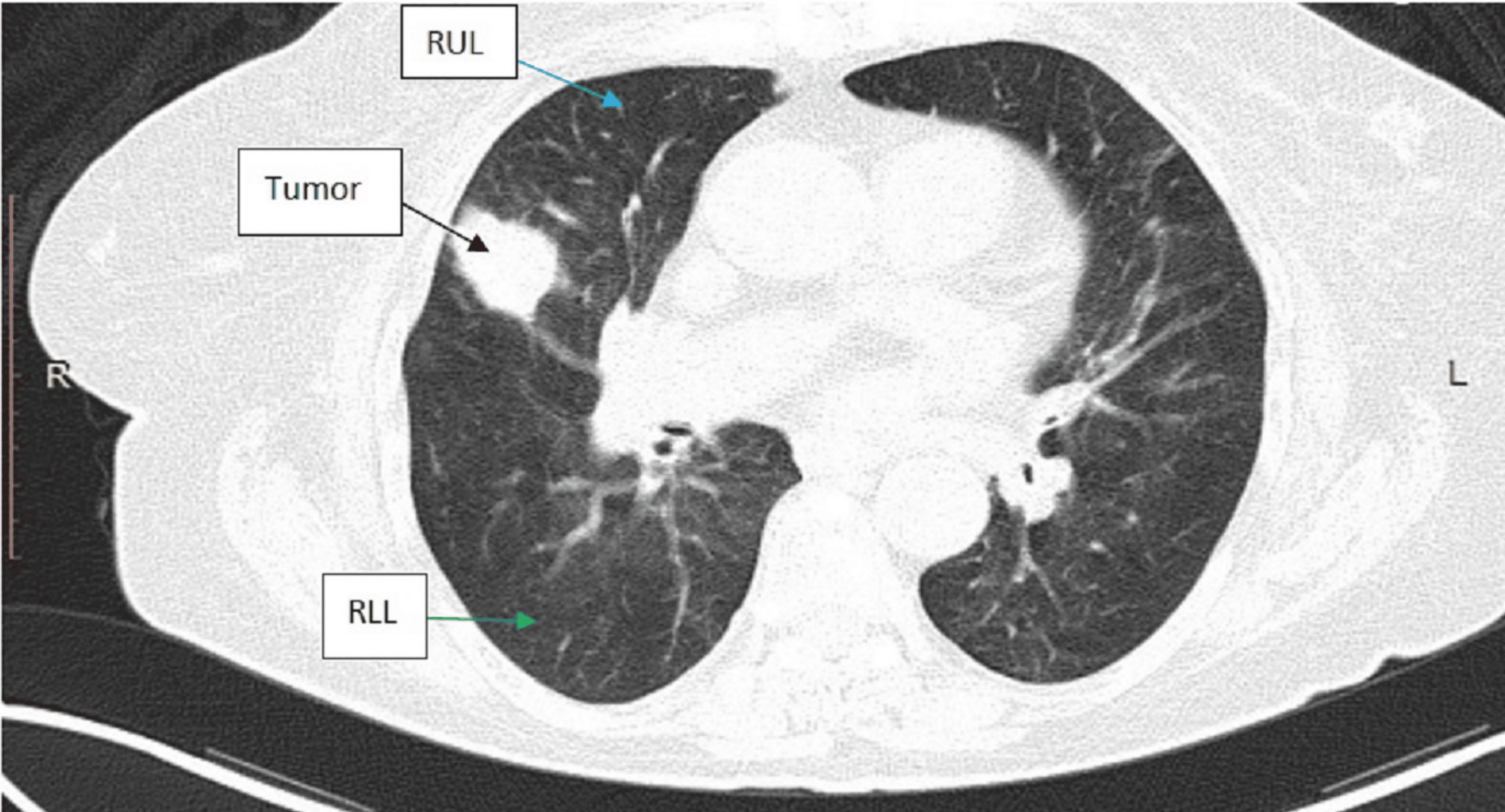
A chest CT revealed a lesion (black arrow) on the posterior segment of the right upper lobe, which was in contact with the minor fissure. RUL: right upper lobe; RLL: right lower lobe

**Figure 2:**
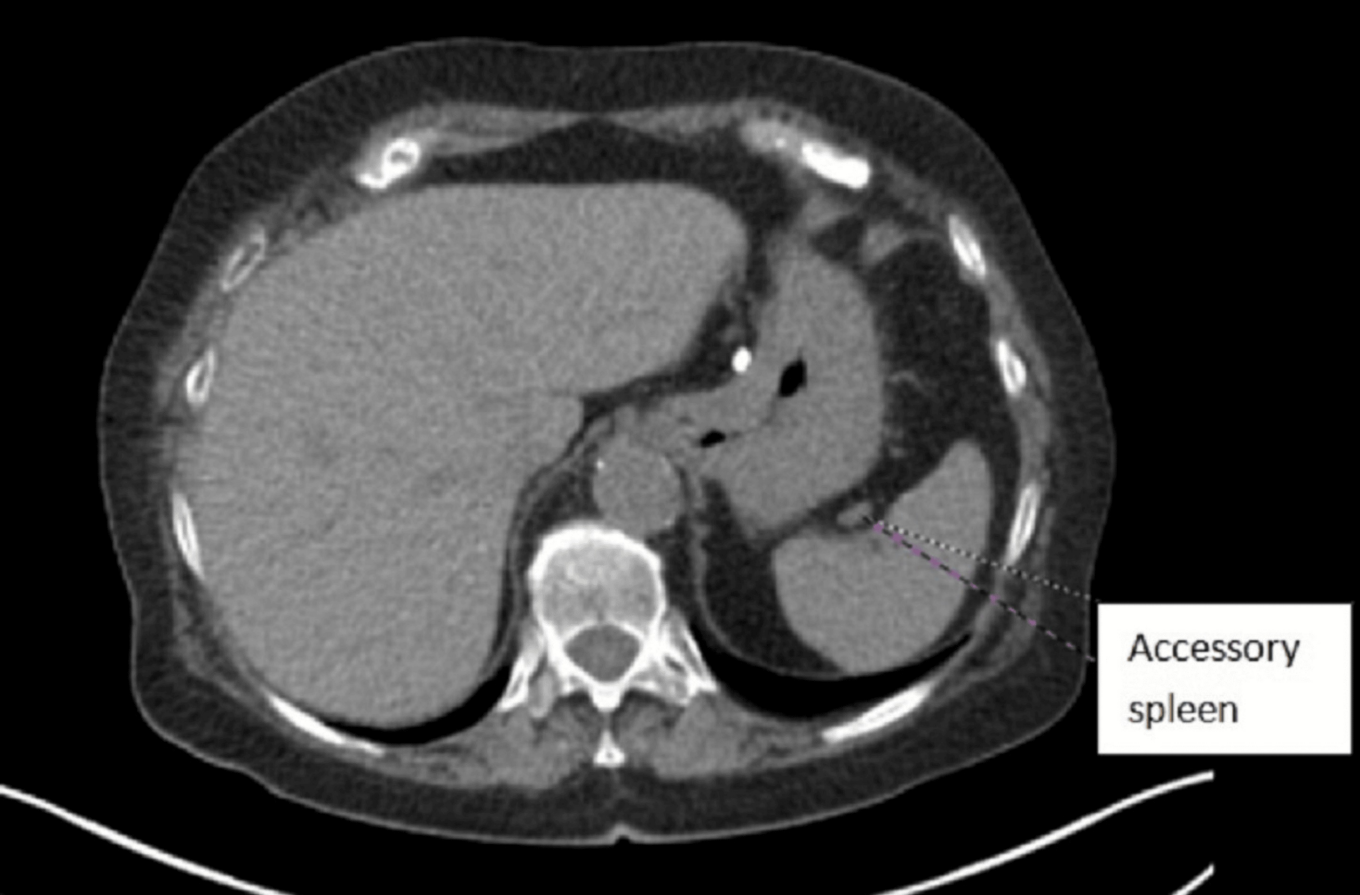
A CT scan of the flattened formation occupying the splenic hilum which was considered as accessory spleen

**Figure 3:**
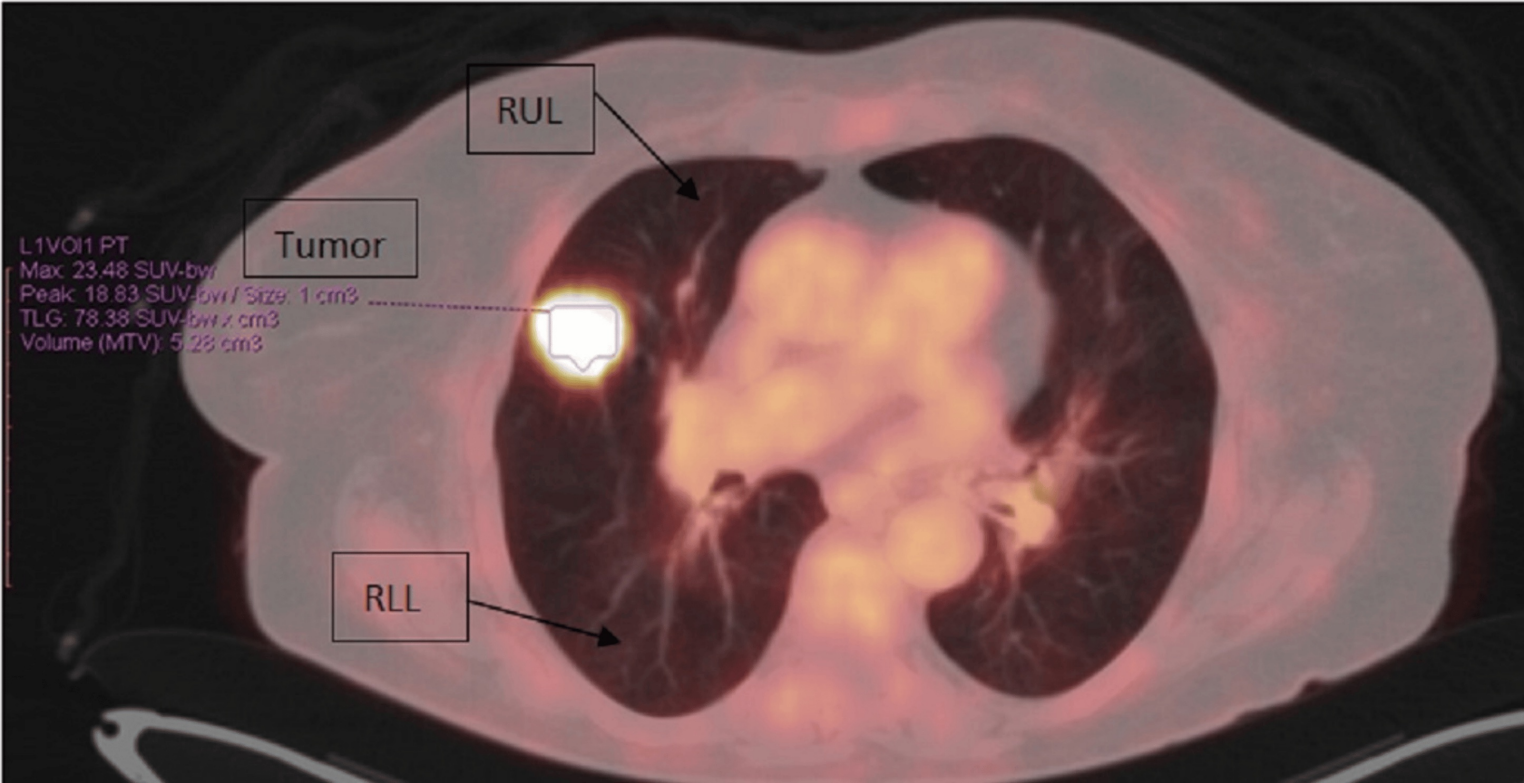
The PET-CT scan showed increased metabolic uptake in the right upper lobe nodule without evidence of hypermetabolic activity in the lymph nodes. RUL: right upper lobe; RLL: right lower lobe

A 78-year-old female patient with a history of diabetes mellitus type II, dyslipidemia, and hypothyroidism due to partial thyroidectomy (40 years ago), surgical removal of vocal cord polyps (20 years ago), and smoking history (120 pack years) was admitted due to fever, with a working diagnosis of respiratory tract infection. A computed tomography (CT) scan of the chest revealed an 18-mm-large (in diameter) nodule in contact with the horizontal fissure on the posterior aspect of the upper lobe of the right lung (Figure 1). The abdominal CT scan revealed two cortical renal cysts, a flattened formation measuring 12 mm in length occupying the splenic hilum (Figure 2), and a diverticulum of the second section of the duodenum. In positron emission tomography-computed tomography (PET-CT), the nodule absorbed 18F-fluorodeoxyglucose (FDG), and the calculated standard unit value (SUV) max was 23.48, raising suspicion of malignancy, whereas the formation of the splenic hilum did not absorb 18F-FDG and was thus considered to be an accessory spleen (Figure 3). No metastatic disease was identified. The patient was referred to the thoracic surgery department, and spirometry (forced expiratory volume in 1 second (FEV1) 1.25 (67% of predicted), diffusing capacity of the lungs for carbon monoxide (DLCO) 65%) was performed. She underwent right thoracotomy, right upper lobectomy (including a small segment of the middle lung lobe), and lymph node dissection.

Upon sections, the pulmonary lobe contained a well-circumscribed grayish tumor with irregular borders of elastic consistency with a maximum diameter of 30 mm. The surgical margins appeared macroscopically uninvolved (10 mm distance from the closest margin). The rest of the lung parenchyma was macroscopically regular. Microscopically, the tumor consisted of large malignant epithelial cells with vesicular nuclear chromatin and conspicuous nucleoli arranged in a syncytial growth pattern; they were admixed with neutrophils and a brisk lymphoplasmacytic infiltrate. Neither lymphovascular invasion nor spread through air spaces was identified. All 11 excised lymph nodes were uninvolved.

**Figure 4:**
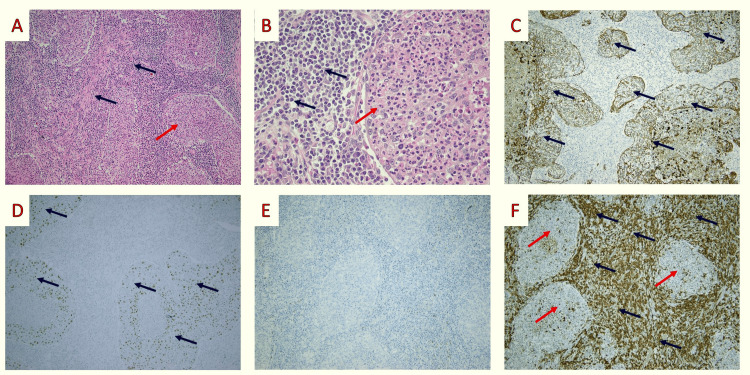
Histopathologic findings: A, B: Histologic evaluation employing hematoxylin-eosin staining revealed large malignant epithelial cells (red arrow), with vesicular nuclear chromatin and conspicuous nucleoli, arranged in a syncytial growth pattern; they were admixed with both neutrophils and a brisk lymphoplasmacytic infiltrate (blue arrows) (hematoxylin and eosin (H&E); × 100 and x400); C, D: Neoplastic cells (blue arrows) exhibited immunopositivity for the CKAE1/AE3 (C) and p40 (D) markers (cytokeratin AE1/AE3 mouse monoclonal AE1/AE3, Dako x100; Dak-p40 rabbit monoclonal, Dako; x 100); E: Tumor cells were negative for TTF-1 (TTF-1 mouse monoclonal 8G7G3/1, Dako); F: Leukocyte common antigen (LCA) highlighted the lymphoplasmacytic infiltrate (blue arrows) while the epithelial cells (red arrows) did not stain (LCA mouse monoclonal 2B11/PD7/26, Dako x 100). Dako: Agilent Technologies, Inc., Santa Clara, CA

Tumor cells were immunostained with cytokeratin (CK) AE1/AE3 and p40 (Figure 4), with no staining for thyroid transcription factor -1 (TTF-1). Leukocyte common antigen (LCA) highlighted the lymphoplasmacytic infiltrate. Epstein-Barr encoding region (EBER)-in situ hybridization was negative. No visceral pleural invasion was documented using an Elastica van Gieson stain (Merck KGaA, Darmstadt, Germany).

Based on the above findings (tumor size, lack of visceral pleural invasion, or lymph node metastasis), a pT1cN0/stage IA3 (according to the American Joint Committee on Cancer (AJCC) 8^th^ edition [18]) lymphoepithelial carcinoma of the lung was diagnosed. On the third postoperative day, the patient experienced an episode of atrial fibrillation/flutter, which was treated with amiodarone and beta-blockers. The patient was discharged in a good physical state and sinus rhythm, with further instructions concerning her heart condition. Ten days after discharge, an initial re-evaluation based on a new chest X-ray was scheduled. Moreover, due to tumor characteristics and early stage (small size, lack of lymph node involvement), the decision of the multidisciplinary team (MDT) meeting was that no adjuvant treatment was required. It was decided to follow up with the patient with a CT scan initially every three months.

## Discussion

Pulmonary lymphoepithelial carcinoma is a distinct subtype of squamous lung carcinoma, frequently associated with EBV infection [2]. Our case differs in some aspects from most cases of similar tumors so far published. Epidemiologically, our patient was a 78-year-old Caucasian smoker, whereas this tumor usually manifests in persons of Asian origin, with a median age of 51 years and, most importantly, nonsmokers. [3,19]. In our case, the carcinoma was an incidental finding discovered during the workup for an upper respiratory tract infection without specific imaging features, similar to other reported cases [20,21].

The histopathological confirmation of lymphoepithelial carcinoma proved challenging. The gross characteristics of the tumor are not specific for this cancer subtype [22]. Based on its histomorphology, the differential diagnosis included metastatic lymphoepithelial carcinomas from other anatomic sites and, most notably, from sinonasal lymphoepithelial carcinoma [23], primary non-Hodgkin lymphoma of the lung, and, most importantly, from marginal zone B-cell lymphoma of mucosa-associated lymphoid tissue (MALT) [24] and NUT carcinoma arising in the lung [25]. In our case, the PET/CT did not reveal metastatic disease, thus excluding the possibility of metastasis from the sinonasal tract. The brisk lymphoplasmacytic stromal infiltration in association with the tumor's syncytial formation helped exclude NUT carcinoma. An experienced hematopathologist rejected the possibility of a MALT lymphoma. Positive staining for the immunohistochemical marker p40 and the tumor's morphological features also favored primary lymphoepithelial carcinoma. Although the association with EBV infection is well established [26, 27], we could not detect it using in situ hybridization. This can be explained by the fact that our patient is Caucasian. Caucasian patients do not typically show an association between EBV infection and pulmonary lymphoepithelial carcinoma [22].

The molecular profile of EBV-related lymphoepithelial lung carcinoma has been thoroughly studied [25, 26]. The driver mutations typically observed in most NSCLC, such as tumor protein p53 (TP53), Kirsten rat sarcoma viral oncogene homolog (KRAS), epidermal growth factor receptor (EGFR), or anaplastic lymphoma kinase (ALK), and ROS proto-oncogene 1 (ROS1) translocations, are rarely present. TNF receptor-associated factor (TRAF3) deletion is usually present [27]. An essential finding with high therapeutic interest is this tumor's remarkable programmed cell death ligand 1 (PD-L1) expression [28-30]. Treatment with immune checkpoint inhibitors might have a favorable outcome [31].

Patients usually seek medical attention while their disease is still limited, meaning early tumor stage and absence of lymph node metastasis [32, 33]. This applied to our patient as well. This fact allows for the complete surgical resection of the tumor. The therapeutic plan can be enhanced with chemotherapy, radiotherapy, or immunotherapy in locally advanced or metastatic disease [4, 17]. The detection at an early stage in most patients is the main reason this specific type of lung carcinoma has a better prognosis compared with other types of NSCLC [32, 33]. Other favorable prognostic factors include the absence of lymph node metastasis, high PDL1 expression or wild-type TP53 expression [32], and normal serum lactate dehydrogenase and albumin levels [16]. Poor prognostic factors include tumor recurrence and the presence of necrosis [34]. 

## Conclusions

Pulmonary lymphoepithelial carcinoma is a poorly differentiated squamous cell lung carcinoma subtype, usually associated with EBV infection. Here, we present a case of a patient with distinct epidemiological features presenting with an early disease stage, thus making complete surgical resection of the tumor possible, a therapy favoring a better disease prognosis.
